# Human Cochlear Histopathology Reflects Clinical Signatures of Primary Neural Degeneration

**DOI:** 10.1038/s41598-017-04899-9

**Published:** 2017-07-07

**Authors:** Jessica E. Sagers, Lukas D. Landegger, Steven Worthington, Joseph B. Nadol, Konstantina M. Stankovic

**Affiliations:** 1 Eaton-Peabody Laboratories, Department of Otolaryngology, Massachusetts Eye and Ear, Boston, MA 02114 United States; 2 Program in Speech and Hearing Bioscience and Technology, Harvard Medical School, Boston, MA 02114 United States; 3 Department of Otolaryngology, Vienna General Hospital, Medical University of Vienna, Vienna, 1090 Austria; 4 Department of Otolaryngology, Harvard Medical School, Boston, MA 02114 United States; 5 Harvard Institute for Quantitative Social Science, Harvard University, Cambridge, MA 02138 USA

## Abstract

Auditory neuropathy is a significant and understudied cause of human hearing loss, diagnosed in patients who demonstrate abnormal function of the cochlear nerve despite typical function of sensory cells. Because the human inner ear cannot be visualized during life, histopathological analysis of autopsy specimens is critical to understanding the cellular mechanisms underlying this pathology. Here we present statistical models of severe primary neuronal degeneration and its relationship to pure tone audiometric thresholds and word recognition scores in comparison to age-matched control patients, spanning every decade of life. Analysis of 30 ears from 23 patients shows that severe neuronal loss correlates with elevated audiometric thresholds and poor word recognition. For each ten percent increase in total neuronal loss, average thresholds across patients at each audiometric test frequency increase by 6.0 dB hearing level (HL). As neuronal loss increases, threshold elevation proceeds more rapidly in low audiometric test frequencies than in high frequencies. Pure tone average closely agrees with word recognition scores in the case of severe neural pathology. Histopathologic study of the human inner ear continues to emphasize the need for non- or minimally invasive clinical tools capable of establishing cellular-level diagnoses.

## Introduction

In individuals with normal hearing, bipolar neurons in the cochlear branch of the eighth cranial nerve project to sensory hair cells in the organ of Corti, relaying precisely timed signals from the spiral-shaped cochlea up the ascending auditory pathway to the brainstem. Damage to the auditory nerve (AN), via demyelination or loss of synapses, axons, or neuronal cell bodies in the spiral ganglion, can result in imprecise temporal coding and a disruption in firing synchrony, yielding a faulty representation of the input signal^1, 2^.

In 1996, Arnold Starr and colleagues coined the term “auditory neuropathy” as a means of classifying patients who demonstrated aberrant responses to assessments of AN function despite normal responses to assessments of sensory cell function^3^. In Starr’s patients, (1) the auditory brainstem response (ABR), an electrophysiological measurement consisting of several peaks, the first of which reflects the summed activity of the AN, was absent or severely distorted; (2) auditory brainstem reflexes, such as the stapedius muscle reflex, were absent; and (3) speech intelligibility scores were disproportionately poorer than expected based on audiometric threshold measurements, though (4) typical measures of outer hair cell (OHC) function, such as otoacoustic emissions and cochlear microphonic potentials, remained within normal limits^3^. These criteria remain the diagnostic hallmarks of auditory neuropathy, though further evaluation via electrocochleography and advanced tests of neural function can refine a diagnosis^4^.

Recent studies suggest that auditory neuropathy is a more prevalent form of hearing loss than initially assumed. Among preterm infants, 1 in every 423 graduates of the newborn intensive care unit meets Starr’s criteria for auditory neuropathy^5^, and among healthy infants, routine screening identifies AN dysfunction in 1 in every 7000^2^. This phenotype has been documented to accompany an array of medical conditions, ranging from developmental dystrophies and genetic mutations^1, 6^ to acquired neuropathies, such as those induced by neoplasm, infection, exposure to noise or drugs, or the deleterious effects of age^4, 7, 8^.

In adults, the prevalence of auditory neuropathy is likely underestimated, because the most widely used diagnostic tool in audiology, the pure tone audiogram, is not capable of predicting cell-type specific damage in the inner ear^9^. Studies of human patients with auditory neuropathy demonstrate that pure tone audiometric thresholds associated with this phenotype can vary from near-normal to highly elevated^4^. Such variation is likely due to the diverse etiology associated with this phenotype. For example, though the synapse between the spiral ganglion neuron (SGN) and inner hair cell (IHC) is the most vulnerable element in the inner ear, human and animal studies historically suggest that damage to the AN and its terminals may not be reflected in traditional measurements of hearing thresholds^10–13^. Indeed, since the 1950s, diffuse damage to the AN without damage to hair cells has been assumed to have little effect on pure tone thresholds^14^.

Because there is currently no way to biopsy or visualize cellular-level structures of the inner ear in living humans, histopathological study of human temporal bones has formed the basis of scientific understanding for numerous auditory pathologies. In 2001, Nadol reviewed primary cochlear neuronal degeneration in individual temporal bones associated with genetic, toxic, immunologic, degenerative, idiopathic, and infectious causes; however, no attempt at collective quantitative analysis was made^8^. To date, five independent studies of temporal bone histopathology in specific forms of hereditary AN dysfunction have been undertaken, all of which involved single patients or small cohorts (n < 5)^1, 15–18^. The consistent finding among these studies was a severe loss of SGNs despite the presence of sensory cell populations within normal limits for age. To place results like these in the context of normal presbycusis, the expected age-related decline in SGNs over the human lifespan, Makary *et al*. charted SGN cell counts from 100 individuals with no known cochlear pathology, generating baseline values for primary neuronal loss against which pathologic degeneration can be compared^19^.

To date, there has been no histological study of pathologic neural degeneration spanning various etiologies. Here, we present hypothesis-driven statistical models of neuronal loss in severe neural degeneration and its relationship to pure tone audiometric thresholds and word recognition scores among 30 ears from 23 patients, spanning every decade of life. Understanding the natural progression of auditory neural degeneration holds important implications for patient counseling and will prove integral for the development of future therapies, as no existing treatment can ameliorate this phenotype.

## Results

### Patients with severe neural degeneration show abnormally low SGN counts with respect to age

**Figure 1:**
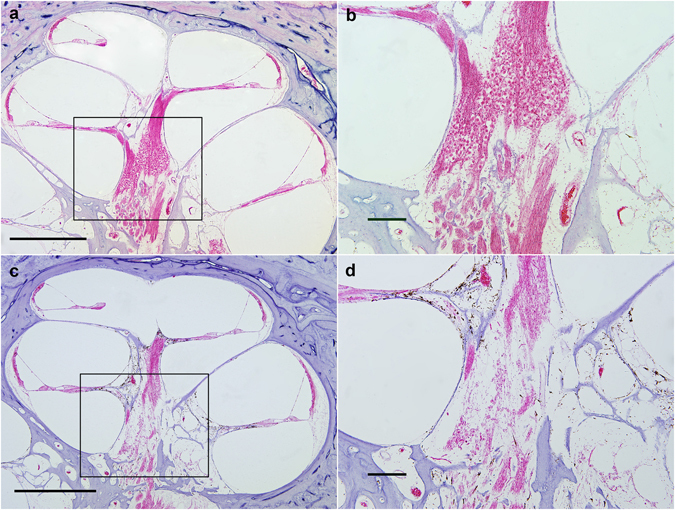
Representative cochlear histopathology in severe primary neural degeneration compared to age-matched control. In both examples, sensory structures are normal. (**a**) mid-modiolar section (4X) from the cochlea of a patient with appropriate SGN numbers for age; scale bar 1 mm. The boxed area is shown magnified in b. (**b**) 10X magnification of the modiolus in a; scale bar 200 μm. (**c**) Mid-modiolar section (4X) from an age-matched patient with severe primary neural degeneration (Patient 19), showing 85% fewer total SGN cell bodies than expected for age; scale bar 1 mm. The boxed area is shown magnified in d. (**d**) 10X magnification of the modiolus in c; scale bar, 200 μm.

Representative cochlear photomicrographs demonstrate the abnormal histopathology characteristic of primary neural degeneration. Sectioning the human cochlea parallel to the modiolus, or conical central axis, reveals Rosenthal’s canal, a spiraling bony channel within the two and a half turns of the cochlea which houses SGN cell bodies. Myelinated peripheral axons fan out from SGNs in this canal, reaching through the osseous spiral lamina to innervate hair cells embedded in the organ of Corti, while central axons project from Rosenthal’s canal to the cochlear nucleus in the brainstem. In humans with no known cochlear pathology, like the 71-year-old male presented in Fig. 1a and b, a large population of neuronal cell bodies is visible in the modiolus. In patients with primary neural degeneration, such as the age- and sex-matched patient presented in Fig. 1c and d, substantially fewer than average SGN cell bodies are observed in the modiolus, though sensory structures appear normal.

**Figure 2:**
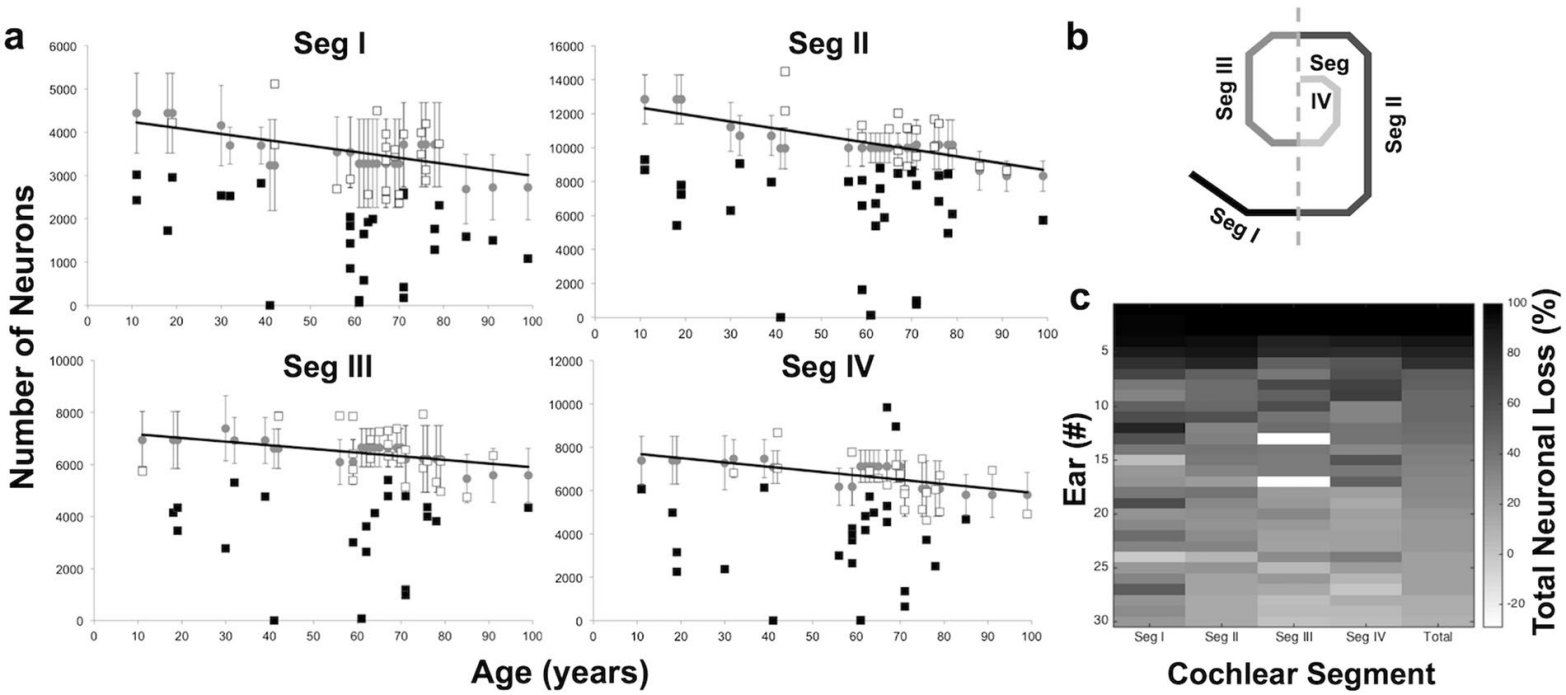
Patients with severe neural degeneration show abnormally low SGN counts with respect to age. SGNs quantified by cochlear segment in 51 ears from 34 patients. (**a**) Gray circles: published age-based mean with standard deviation and linear regression (Makary *et al*., 2011); black squares: patients with SGN counts greater than one standard deviation below the age-based mean; white squares: all other patients. (**b**) Schematic of cochlear segments, as in Makary *et al*., 2011. (**c**) Heat map of 30 ears from 23 patients exhibiting total neuronal loss (%) greater than one standard deviation below the age-based mean; each line represents a single ear.

In the normally aging human population, SGN counts are known to demonstrate a clear decline per decade of life, with no significant gender or inter-aural differences^19, 20^. Here, SGN cell bodies in 51 ears from 34 patients identified as candidates for inclusion in this study were quantified and compared with the age-based mean for normal SGN cell death expected for decade of life, published by Makary *et al*.^19^ (Fig. 2a). SGNs are traditionally quantified by splitting the cochlear spiral into four segments of relatively equal length, in order to capture the critical apical-to-basal gradient (Fig. 2b). After segment-specific quantification of SGN cell bodies in all identified patients, 30 ears from 23 patients demonstrated total SGN counts greater than one standard deviation below the mean SGN count expected for decade of life given normal presbycusis (Fig. 2c). Clinical characteristics for these patients, including otologic diagnoses and cause of death, are summarized in Table 1.

**Table 1:** Summary of clinical characteristics among patients with SGN counts greater than one standard deviation below the mean expected for age, despite a normal complement of sensory cells (n = 30 ears belonging to 23 patients).

Patient	Age	Sex	Ear	Primary otologic diagnosis	Cause of death
1	11	F	R	Neuroaxonal dystrophy	Respiratory arrest
			L		
2	18	M	L	Severe neural degeneration	Craniopharyngioma
3	19	M	R	Fibrous sclerosis of middle ear	Renal failure
			L		
4	30	M	R	Sclerosteosis	Postoperative complications of cranial decompression
5	32	M	R	Histiocytosis	Thyroid carcinoma with metastasis
6	39	M	R	Exostosis of external auditory canal	Malignant lymphoma
7	41	F	R	Severe atrophy of cochlear nerve, etiology undetermined	Epipharyngeal carcinoma
8	56	M	R	Presbycusis	Bacterial endocarditis
9	59	F	R	Acute otitis media	Intracranial hemorrhage
10	59	M	L	Hypoplastic dysmorphic modiolus	Ruptured aortic aneurysm
11	61	M	R	Mohr-Tranebjaerg syndrome	Unknown
			L		
12	62	M	R	Chronic otitis media	Unknown
			L		
13	63	M	L	Otosclerosis	Intracranial hemorrhage
14	64	M	L	Primary neural degeneration	Chronic respiratory insufficiency
15	67	M	R	High-tone hearing loss	Acute urinary failure
16	67	M	L	Presbycusis	Hepatic failure with gastrointestinal hemorrhage
17	70	M	R	Otosclerosis	Malignant lymphoma
			L		
18	71	F	L	Primary neural degeneration	Intracerebral hemorrhage
19	71	M	R	Neural presbycusis	Necrotizing bronchopneumonia
			L		
20	76	F	R	Presbycusis	Coronary thrombosis
21	78	M	R	Presbycusis	Cardiopulmonary arrest
			L	Meniere’s, presbycusis	
22	79	M	R	Presbycusis	Cardiovascular accident
23	99	F	L	Presbycusis	Unknown

Patients listed from youngest to oldest. Sex, F(emale) or M(ale); ear: R(ight) or L(eft).

**Figure 3:**
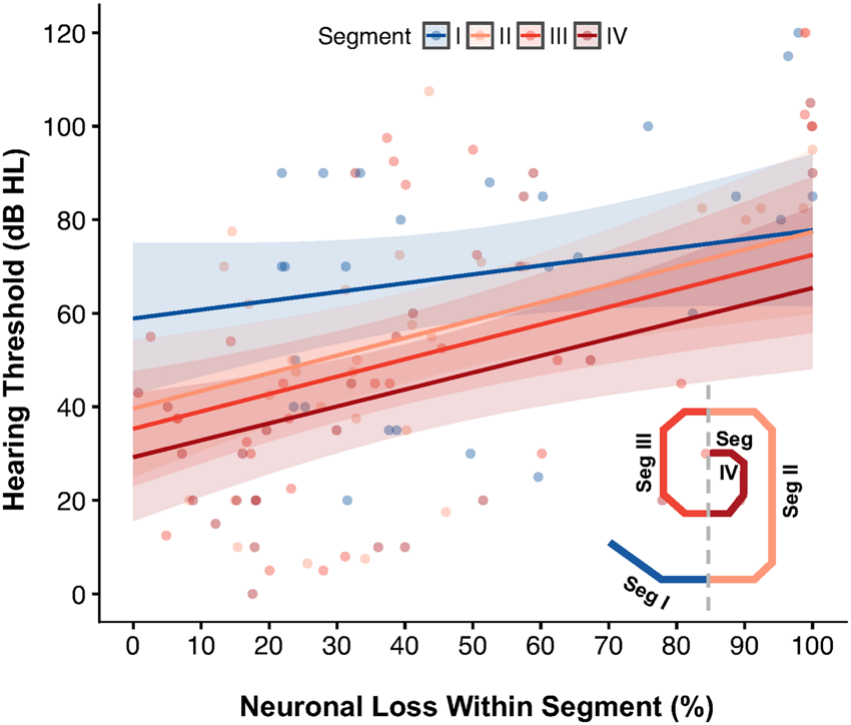
Relationship between hearing thresholds (dB HL) and total neuronal loss (% of age-based mean) does not vary significantly across cochlear length within patients with severe neural degeneration (n = 30 ears). Cochlear length analyzed in four segments (see inset). Solid lines, linear regressions per cochlear segment; shaded areas, 95% confidence intervals for slope; data points, individual observations color-coded by cochlear segment. Conditional f test with Kenward-Rogers correction for degrees of freedom shows that the slopes of these lines are significantly different from zero (p < 0.001), but not significantly different from one another (p = 0.43).

Among the 30 ears with severe primary neuronal degeneration, neuronal loss in Segment II, which encodes frequencies between 1.2–8 kHz, mimicked total neuronal loss most closely (Fig. 2c). Substantial threshold elevation was more likely to accompany neuronal loss in Segment I than loss in any other segment, as reflected in the y-intercept (p = 0.02 when compared with y-intercept for Seg II; p < 0.001 when compared with y-intercepts for Segs III–IV; sequential Bonferroni-adjusted for multiple comparisons). However, a linear mixed effects regression model showed that the rate of increase in hearing thresholds with total neuronal loss in Segment I was not significantly different than that exhibited in any other cochlear segment (p = 0.43) (Fig. 3).

Segment I corresponds with the cochlear base, a region particularly vulnerable to myriad forms of damage throughout the lifetime^21^. Even when inner hair cells are intact, normally aging cochleae often demonstrate significant outer hair cell loss in the basal turn, which contributes to elevated high-frequency thresholds common in older adults^22, 23^. To quantify the effect of hair cell loss on threshold elevation, cytocochleograms reporting the presence or absence of individual hair cells in each cochlear segment were examined. Cytocochleograms were available for ten of the 30 ears included in this analysis; however, mean reported hair cell loss was negligible in each cochlear segment (Seg I, 2.3% of hair cells lost; Seg II, 2.8%; Seg III, 0.4%; Seg IV, 3.4%). Given only ten observations, hair cell loss could not be reliably controlled for in the existing model. However, nontrivial threshold elevation in Segment I, even in the case of neuronal loss no different than is typical for age, suggests the contribution of other cell types to the observed high-frequency hearing loss.

### Primary neuronal loss correlates significantly with elevated audiometric thresholds and poor word recognition

**Figure 4:**
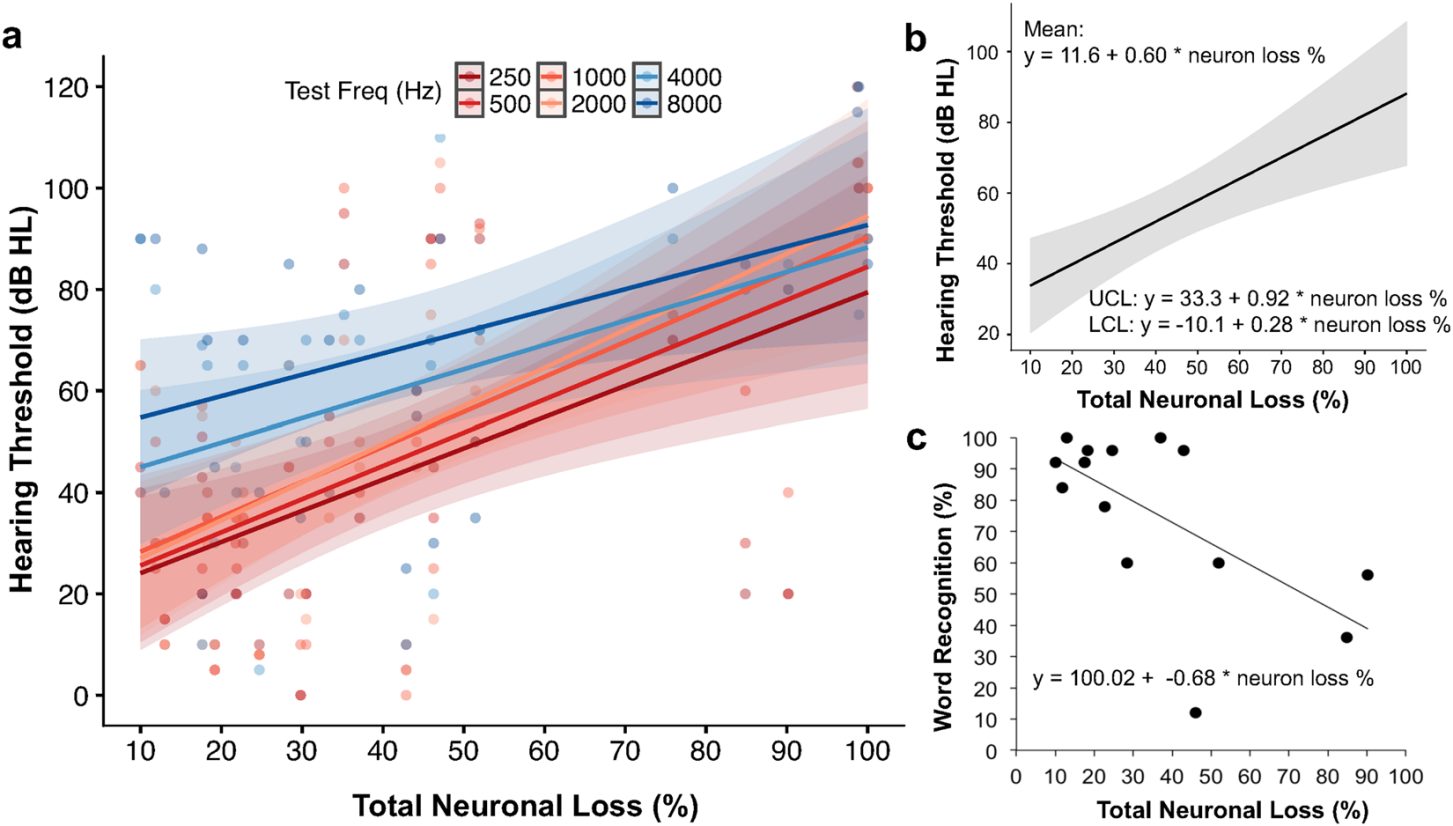
Primary neuronal loss correlates significantly with elevated audiometric thresholds and poor word recognition scores. Confidence intervals are represented as shaded areas around regression lines and are measures of 95% precision for predicted slopes and intercepts. (**a**) Linear mixed regression model correlating total neuronal loss (% of age-based mean) with audiometric thresholds (dB HL) within patients at each individual test frequency (n = 30 ears). Solid lines, linear regressions per audiometric test frequency (Freq); data points, individual threshold observations color-coded by audiometric test frequency. Conditional f test with Kenward-Rogers correction for degrees of freedom reveals that slopes of these lines are significantly different than zero (p < 0.001), but not significantly different from one another (p = 0.10). (**b**) Averaging across all six audiometric test frequencies yields a mean threshold increase of 6.0 dB HL per 10% total neuronal loss; shaded area, 95% confidence interval. (**c**) Total neuronal loss (% of age-based mean) correlates with poor word recognition (n = 15 ears). Mean word recognition score decreases by 6.8% per 10% total neuronal loss (r = −0.644).

In 30 ears from 23 patients with severe neural degeneration, a linear mixed effects regression model was used to correlate audiometric thresholds at each of six individual audiometric test frequencies with total neuronal loss as a percentage of the mean expected for age (Fig. 4a). For each ear, all recorded hearing thresholds, color-coded to represent the audiometric test frequency at which each threshold was recorded (0.25–8 kHz), were graphed in a vertical line along the y-axis at the single point along the x-axis representing the percentage of total neuronal loss observed in that patient. Regression lines and 95% confidence intervals for slope were then calculated and compared across thresholds recorded at each individual audiometric test frequency (Supplementary Fig. S1). In this way, overall relationships among patients for thresholds recorded at each audiometric test frequency can be examined as a function of total neuronal loss.

A significant increase in hearing thresholds was observed as neuronal loss increased over all six audiometric test frequencies (p < 0.001). As observed in Fig. 3, the model suggests a qualitatively different relationship between the rate of threshold increase with neuronal loss at higher audiometric test frequencies and that observed at lower frequencies; however, omnibus tests for differences in slope, corrected for multiple comparisons, show that the hypothesis of a differing relationship among these lines approaches, but does not reach statistical significance (p = 0.10). Predicted means and Bonferroni-adjusted contrasts at each audiometric test frequency per ten percent neuronal loss are provided in Supplementary Tables S1 and S2. As the rate of increase in hearing threshold over total neuronal loss does not significantly differ among audiometric test frequencies, these relationships can be collapsed into a single linear model (Fig. 4b). In this way, averaging across all six audiometric test frequencies yields a mean threshold increase of 6.0 dB HL per 10% total neuronal loss.

Word recognition scores, reported as a percentage of correctly repeated words after being read a word list in quiet, were available for 15 of 30 ears of interest (12 patients). Word recognition among these patients declined significantly as total neuronal loss increased (Fig. 4c). Linear regression revealed a 6.8% decline in word recognition score per 10% increase in total neuronal loss (r = −0.644).

### Thresholds relevant to word recognition rise with neuronal loss among patients with severe neural degeneration

**Figure 5:**
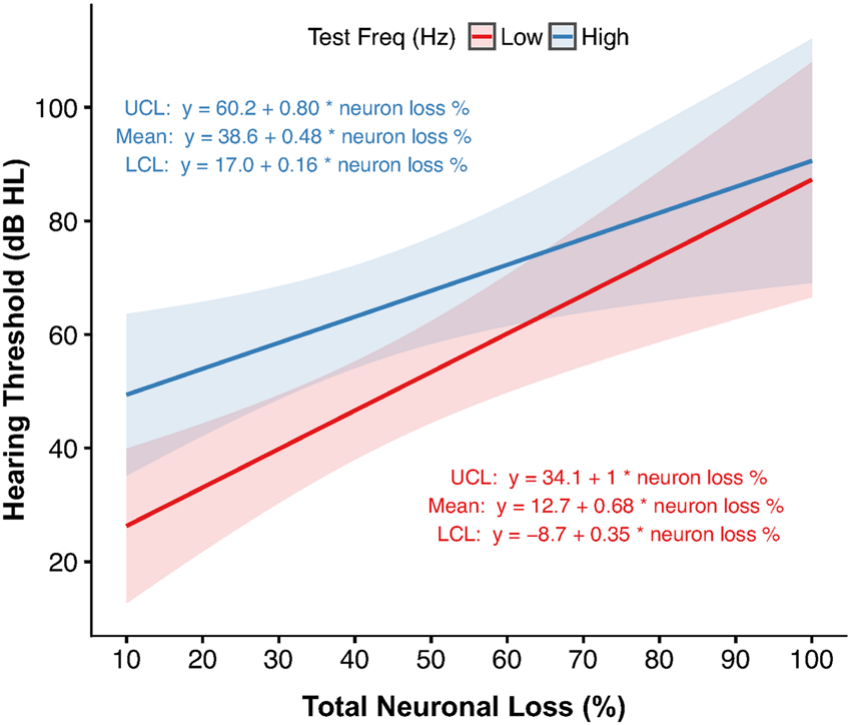
High-low grouping of audiometric test frequencies reveals a significant interaction between total neuronal loss (% of age-based mean) and hearing thresholds (dB HL) (n = 30 ears). Low frequencies, 0.2–2 kHz (6.8 dB HL mean threshold increase per 10% total neuronal loss); high frequencies, 4–8 kHz (4.8 dB HL mean threshold increase per 10% total neuronal loss); p = 0.007. Solid lines, linear regressions per low or high frequency grouping; shaded areas, 95% confidence intervals. UCL, upper confidence limit; LCL, lower confidence limit.

To further explore the observation that elevated thresholds at high frequencies may reflect the involvement of factors outside neuronal pathology (Fig. 3), threshold data was grouped into that belonging to low frequencies (0.25–2 kHz) and high frequencies (4–8 kHz) and regressed with regard to total neuronal loss using a linear mixed effects regression model (Fig. 5). Such classification is justified as low frequencies predominantly influence word recognition^24, 25^. In fact, on a clinical audiogram, normal hearing thresholds at frequencies up to and including 2 kHz are covered by the belly of a down-sloping area colloquially referred to as the “speech banana”^26, 27^. Among low-frequency observations, mean hearing thresholds increase by 6.8 dB HL per 10% total neuronal loss, while among high-frequency observations, mean hearing thresholds increase by 4.8 dB HL per 10% total neuronal loss. The difference in rate of threshold increase between low-frequency and high-frequency observations is statistically significant (p = 0.007). As in Fig. 4a, 95% confidence intervals for the low- and high-frequency groupings exhibit little overlap below 40% total neuronal loss, after which they share significant overlap, becoming nearly equivalent as loss approaches 100% (Supplementary Table S3). Additionally, as observed in Fig. 4a, a significant elevation in the y-intercept for high-frequency observations, apparent even without corresponding neuronal loss, highlights the contribution of threshold-elevating factors that lie outside the influence of neuronal loss.

**Figure 6:**
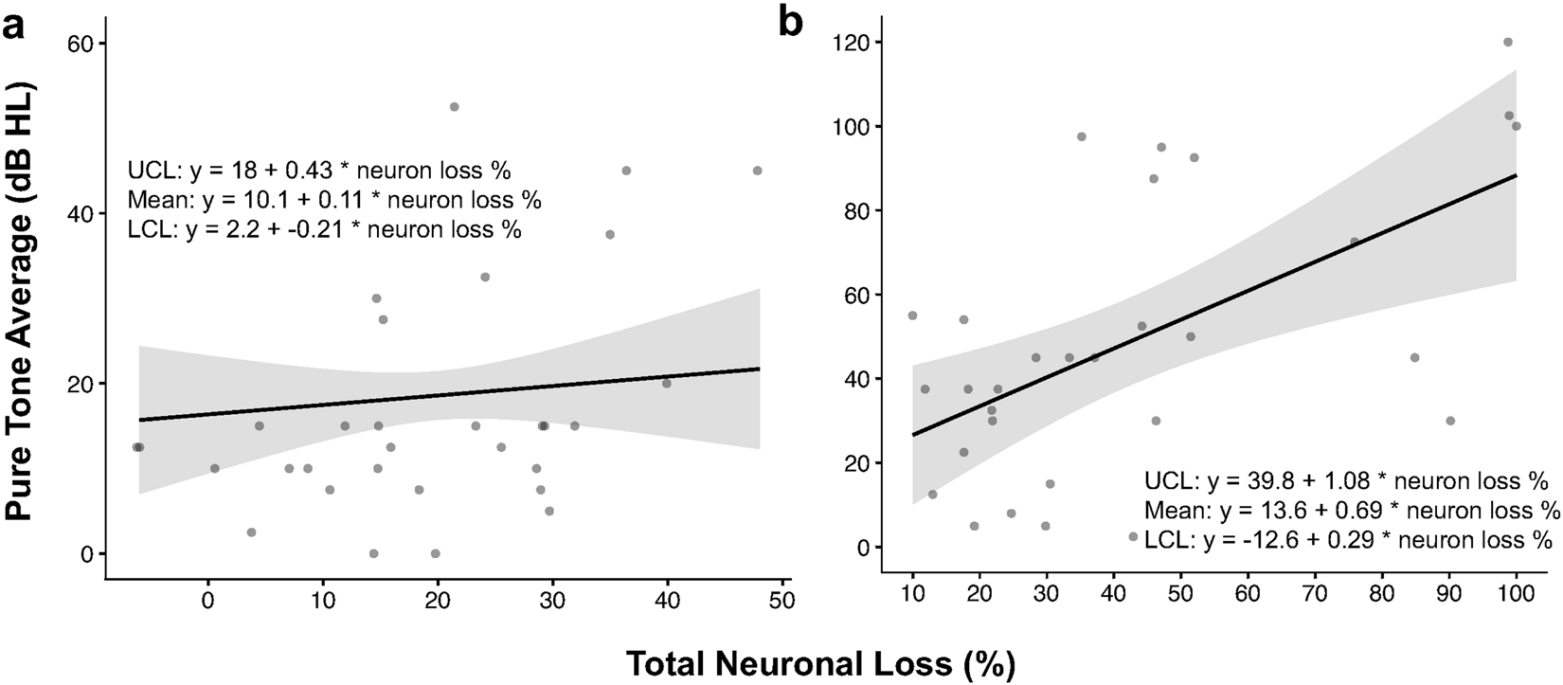
Relationship between pure tone average (dB HL) and total neuronal loss (% of age-based mean) in severe neural degeneration. (**a**) Normal presbycusis (n = 33 ears, published in Makary *et al*., 2011); (**b**) severe neural degeneration (n = 30 ears). In patients with severe neural degeneration, mean hearing thresholds increase by 6.9 dB HL per 10% total neuronal loss. Shaded area, 95% confidence interval; UCL, upper confidence limit; LCL, lower confidence limit.

Pure tone average (PTA), defined as the average of the two or three lowest thresholds at audiometric test frequencies between 0.5–2 kHz, is used in the otology clinic as a reliable approximation of speech reception and speech detection thresholds^24, 28^. A linear mixed effects regression model was used to describe the relationship between pure tone average and total neuronal loss as a percentage of the age-based mean in normal presbycusis (33 ears, published in Makary *et al*., 2011; Fig. 6a) and ears with severe neural degeneration (30 ears; Fig. 6b). After controlling for age, sex, and time between most recent audiogram and death, PTA rises with neuronal loss among patients with severe neuronal loss, but not among patients with normal presbycusis, suggesting that the threshold elevation accompanying this loss must be attributable to factors other than normal aging. However, the variance in both of these datasets is too large to indicate a statistically significant difference and warrants future study involving more patients. As expected based on the “low” frequency grouping in Fig. 4, mean PTA among patients with severe neural degeneration is observed to increase by an average of 6.9 dB HL per 10% total neuronal loss.

## Discussion

We present the largest quantitative study to date of severe neural degeneration in the human inner ear. As cellular structures within the cochlea cannot be biopsied or visualized during life, approaching auditory neuropathy from a histopathological perspective provides important evidence for the cellular substrates of clinical assessments. Our model suggests that mean hearing thresholds in ears with severe primary neural degeneration increase by 6.0 dB HL per 10% total neuronal loss (Fig. 4b). This is a new and highly significant quantitative relationship.

However, though the positive relationship between these variables is clear, a simple linear increase in hearing thresholds is not well explained by animal models of excitotoxic, physical, or drug-induced neuropathy. For example, after noise exposure in mice, Kujawa and Liberman observed a temporary elevation of audiometric thresholds followed by a return to normal values, despite widespread synaptopathy^11^. Schuknecht and Woellner, who pierced the auditory nerves of cats with needles to damage up to 50% of AN fibers, observed only mild effects of this trauma on a behavioral audiogram^29^. More recently, Lobarinas *et al*. used carboplatin to induce large amounts of IHC death in trained chinchillas, observing aberrant responses in the behavioral audiogram only when IHC loss exceeded 80%^30^. However, significant threshold elevation is known to accompany multiple forms of auditory neuropathy regardless of hair cell loss in humans, including cochlear nerve hypoplasia, a diagnosis of gradable severity thought to represent most cases of unilateral auditory neuropathy in humans^6, 7^.

Importantly, despite the fact that the hair cells of patients presented in this study are morphologically intact and present in numbers normal for age, no evidence exists as to whether or not these cells were functional in life. It is well known that functional and chemical changes can long precede morphologic changes in the cochlea^11, 31^. Though the defining characteristic of this patient population is severe neural degeneration, diseases thought to be primarily neurodegenerative often affect the function of and communication between neighboring supporting or interconnected cells that form corresponding parts of a less-than-functional system^32^.

In everyday life, the most significant functional consequence for a patient with auditory neuropathy is poor speech perception, regardless of whether he or she demonstrates elevated audiometric thresholds^7^. A classic complaint from such a patient is that they “can hear, but cannot understand,” and this claim is documented in the written medical records of at least two patients included in this study. For these patients, perceiving speech in noisy environments can be a nearly insurmountable task, perhaps due to loss of SGNs with low spontaneous firing rates and high sound thresholds, which confer important dynamic range to the response patterns of the auditory nerve and are selectively lost as a result of traumatic noise exposure^33^. In our model, the slope of the regression line representing decline in word recognition with increasing neuronal loss is −0.68 (Fig. 4c), a slope opposite and nearly identical to that for PTA with increasing neuronal loss (+0.69, Fig. 6). This is an important finding, as PTA closely agrees with speech reception thresholds^24, 25^ even in the case of severe neural pathology.

Our models have implications for patient counseling. If a patient presents with elevated PTA, demonstrates poor word recognition, and complains of difficulty hearing speech in noise, auditory neuropathy should be included in a differential diagnosis. After functional testing of the AN and OHCs to confirm the diagnosis, if severe degeneration is suspected, total neuronal loss could potentially be approximated using PTA (Fig. 6). This is relevant for timing of a possible cochlear implant surgery, which relies on an array of intracochlearly inserted electrodes to electrically stimulate the remaining neurons. Word recognition is currently the most important criterion for assessing cochlear implant candidacy, by which patients scoring below 60% qualify for this prosthesis. Our data suggest that for people with severe primary neuronal degeneration, the 40% drop in word recognition that precedes cochlear implant candidacy corresponds with a 58.8% loss of SGNs. This is important for patient counseling because auditory rehabilitation after cochlear implantation is correlated with the number of surviving cochlear neurons^34^. Our findings also highlight the need to develop clinically relevant imaging tools that enable cellular-level resolution of structures within the inner ear^35, 36^, as there is currently no way to confirm a diagnosis of primary neural degeneration or verify predicted loss *in vivo*.

An important limitation of this study is the fact that our models do not account for damage to the stria vascularis, a segment of stratified epithelium in the cochlear duct that contains small blood vessels and is responsible for maintaining the endocochlear potential. Much like neural presbycusis, damage to the stria vascularis accumulates over time and has been documented to contribute to hearing loss and metabolic presbycusis in humans and animals^37–39^. However, unlike neural presbycusis, there has been no comprehensive, age-based quantification of expected strial pathology in humans against which to make reliable comparisons.

Additionally, though postmortem quantification of SGN cell bodies in the cochlea has been traditionally used to assess AN viability, such quantification may not yield the most accurate representation of the functional consequences of AN pathology^31^. The synapse between the SGN and IHC is the most vulnerable element in the inner ear and can be irreparably lost after transient acoustic trauma^11^, but SGN cell bodies can take decades to die, surviving even following loss of their peripheral axons^40^. This observation is important when considering potential therapeutic approaches to acquired forms of auditory neuropathy. If the failure of lost synapses to regenerate after acoustic trauma can be attributed to impaired neurotrophic signaling in the organ of Corti^41^, viral overexpression of important neurotrophic factors, such as neurotrophin 3, has the potential to successfully regenerate these synapses, as recently shown^42^. In that case, the fact that SGN cell bodies remain alive in the modiolus decades after losing functional connections to sensory cells provides a fascinating avenue for therapeutic innovation.

## Methods

All human temporal bones analyzed for this study were stored in the archival collection at Massachusetts Eye and Ear and handled according to protocols approved by the associated Human Studies Committee. Informed consent regarding participation in the National Temporal Bone Registry was obtained from all individuals prior to death. Sample collection and human specimen storage was conducted in accordance with Massachusetts Eye and Ear protocols and the Helsinki Declaration. Temporal bones were fixed in 10% neutral buffered formalin or Heidenhain Susa solution, decalcified in ethylenediaminetetraacetic acid, embedded in celloidin, and serially sectioned (20 μm) according to published protocols^20^. Every tenth section was mounted on a glass slide, stained with hematoxylin and eosin, and visualized under a light microscope. 51 bones from 34 patients identified for inclusion in this study (age range 11–99 years, median 67 years) were documented to have a population of IHCs and OHCs normal for age as verified by light microscopy, and a qualitatively abnormal population of spiral ganglion neurons, which we subsequently quantified. Cytocochleograms were available for ten of 30 patients.

SGN counts were conducted according to the method originally described by Schuknecht and followed by subsequent investigators^19, 20, 43^. In every tenth section of each cochlear turn, SGNs in which a nucleus was visible were manually counted. Total neuronal counts per cochlea were then estimated by multiplying SGN counts by 10 to account for intervening sections, and by 0.91 to correct for dual-counting of nuclei spanning section boundaries, in accordance with the most recent recommendations^44^. Totals were compared to published reference values for total cochlear neuronal populations observed in age-matched patients with typical age-related cochlear neuronal degeneration^19^. Bones with total neuronal counts more than one standard deviation below the expected mean for normal, age-related cochlear neuronal degeneration (30 ears from 23 patients) were included in subsequent linear regression models. Otologic diagnoses recorded for these patients varied (Table 1), ranging from neuronal atrophy or advanced presbycusis (16 ears, 14 patients) to near-complete auditory neuropathy due to Mohr-Tranebjaerg syndrome (2 ears, 1 patient).

The most recent clinical audiogram recorded prior to the death of each patient, comprising pure tone detection thresholds at 250, 500, 1000, 2000, 4000, and 8000 Hz, was included in regression models. Pure tone average (PTA), defined at our institution as the average of the two lowest pure tone thresholds at test frequencies between 0.5 and 2 kHz^24, 28^, was also noted. No significant differences to any model parameters were observed when a three-point average (average of pure tone thresholds at 0.5, 1, and 2 kHz) was used. Word recognition scores, recorded as a percentage of correctly repeated words after being read a word list in quiet, were available for 15 of 30 ears (12 patients) of interest.

Linear mixed effects regression models were used to estimate the relationship between audiometric test frequencies and neuronal damage as a percentage of the published age-based mean^19^, in total and after dividing the cochlear spiral into four segments of relatively equal length. All models included controls for linear age, sex, and time interval between each patient’s most recent audiogram and date of death. For omnibus tests of differences in slope, conditional F-tests with Kenward-Rogers correction for degrees of freedom were performed. Computations were carried out and graphs generated in R^45^. For all analyses, p < 0.05 was considered statistically significant.

### Data Availability

The temporal bones and patient records analyzed in the current study are publicly available to trained researchers and can be accessed through the National Temporal Bone, Hearing, and Balance Pathology Resource Registry at Massachusetts Eye and Ear, established by the National Institute on Deafness and Other Communication Disorders (NIDCD) of the National Institutes of Health (NIH).

## Electronic supplementary material

Supplementary PDF File

## References

[1] Starr A (2003). Pathology and physiology of auditory neuropathy with a novel mutation in the MPZ gene (Tyr145->Ser). Brain..

[2] Rance, G. & Starr, A. Auditory neuropathy/dys-synchrony in *Comprehensive Handbook of Pediatric Audiology* (eds Seewald, R. & Tharpe, A. M.) 225–42 (Plural Publishing, 2011).

[3] Starr A, Picton TW, Sininger Y, Hood LJ, Berlin CI (1996). Auditory neuropathy. Brain..

[4] Moser T, Starr A (2016). Auditory neuropathy—neural and synaptic mechanisms. Nat Rev Neurol.

[5] Rance G (2006). Clinical findings for a group of infants and young children with auditory neuropathy. Ear Hear..

[6] Buchman CA (2006). Auditory neuropathy characteristics in children with cochlear nerve deficiency. Ear Hear..

[7] Starr, A. & Rance, G. Auditory neuropathy in *Handbook of Clinical Neurology* vol. 129, series 3, *The Human Auditory* System (eds Celesia, G. G. & Hickok, G.) (2015).

[8] Nadol, J. B. Primary cochlear neuronal degeneration in *Auditory neuropathy: a new perspective on hearing disorders* (eds Sininger, Y. & Starr, A.) 99–140 (Singular, 2001).

[9] Landegger LD, Psaltis D, Stankovic KM (2016). Human audiometric thresholds do not predict specific cellular damage in the inner ear. Hear Res..

[10] Liberman MC, Epstein MJ, Cleveland SS, Wang H, Maison SF (2016). Toward a differential diagnosis of hidden hearing loss in humans. PLoS One.

[11] Kujawa SG, Liberman MC (2009). Adding insult to injury: cochlear nerve degeneration after “temporary” noise-induced hearing loss. J Neurosci..

[12] Schaette R, McAlpine D (2011). Tinnitus with a normal audiogram: physiological evidence for hidden hearing loss and computational model. J Neurosci..

[13] Jensen JB, Lysaght AC, Liberman MC, Qvortrup K, Stankovic KM (2015). Immediate and delayed cochlear neuropathy after noise exposure in pubescent mice. PLoS One..

[14] Schuknecht HF, Woellner RC (1953). Hearing losses following partial section of the cochlear nerve. Laryngoscope..

[15] Spoendlin H (1974). Optic cochleovestibular degenerations in hereditary ataxias. II. Temporal bone pathology in two cases of Friedreich’s ataxia with vestibulo-cochlear disorders. Brain..

[16] Hallpike CS, Harriman DG, Wells CE (1980). A case of afferent neuropathy and deafness. J Laryngol Otol.

[17] Merchant SN (2001). Temporal bone histopathologic and genetic studies in Mohr-Tranebjaerg syndrome (DFN-1). Otol Neurotol.

[18] Bahmad F, Merchant SN, Nadol JB, Tranebjaerg L (2007). Otopathology in Mohr-Tranebjaerg syndrome. Laryngoscope.

[19] Makary CA, Shin J, Kujawa SG, Liberman MC, Merchant SN (2011). Age-related primary cochlear neuronal degeneration in human temporal bones. JARO.

[20] Merchant, S. N. Methods of removal, preparation and study in *Schuknecht’s Pathology of the Ear*, *ed*. *3* (eds Merchant, S. N. & Nadol, J. B.). (Shelton, 2010).

[21] Wong ACY, Ryan AF (2015). Mechanisms of sensorineural cell damage, death and survival in the cochlea. Frontiers in Aging Neuroscience.

[22] Johnsson LG, Hawkins JE (1972). Sensory and neural degeneration with aging, as seen in microdissections of the human inner ear. Ann Otol Rhinol Laryngol.

[23] Perez P, Bao J (2011). Why do hair cells and spiral ganglion neurons in the cochlea die during aging?. Aging Dis.

[24] Fletcher, H. *Speech and Hearing in Communication* (Van Nostrand, 1953).

[25] O’Neil, E., Thornton, A. & Hou, Z. Clinical use of the articulation index in evaluating candidacy for amplification. *Int Hear Aid Conf II* (1995).

[26] Vogel DA, McCarthy PA, Bratt GW, Brewer C (2007). The clinical audiogram: its history and current use. Communicative Disorders Review.

[27] Cherry R (1997). An integrated approach to aural rehabilitation. Seminars in Hearing.

[28] West JS, Low JC, Stankovic KM (2016). Revealing hearing loss: a survey of how people verbally disclose their hearing loss. Ear Hear.

[29] Woellner RC, Schuknecht HF (1955). Hearing loss from lesions of the cochlear nerve: an experimental and clinical study. Trans Am Acad Ophthalmol Otolaryngol.

[30] Lobarinas E, Salvi R, Ding D (2013). Insensitivity of the audiogram to carboplatin induced inner hair cell loss in chinchillas. Hear Res..

[31] Viana LM (2015). Cochlear neuropathy in human presbycusis: confocal analysis of hidden hearing loss in post-mortem tissue. Hear Res..

[32] Garden GA, La Spada AR (2012). Intercellular (mis) communication in neurodegenerative disease. Neuron.

[33] Furman AC, Kujawa SG, Liberman MC (2013). Noise-induced cochlear neuropathy is selective for fibers with low spontaneous rates. J Neurophysiol..

[34] Seyyedi M, Viana LM, Nadol JB (2014). Within-subject comparison of word recognition and spiral ganglion cell count in bilateral cochlear implant recipients. Otol Neurotol.

[35] Yang X (2013). Two photon microscopy of the mouse cochlea *in situ* for cellular diagnosis. J Biomed Optics.

[36] Iyer JS (2016). Micro-optical coherence tomography of the mammalian cochlea. Sci Rep.

[37] Ohlemiller KK, Lett JM, Gagnon PM (2006). Cellular correlates of age-related endocochlear potential reduction in a mouse model. Hear Res..

[38] Pauler M, Schuknecht HF, White JA (1988). Atrophy of the stria vascularis as a cause of sensorineural hearing loss. Laryngoscope..

[39] Dubno JR, Eckert MA, Lee F-S, Matthews LJ, Schmiedt RA (2013). Classifying human audiometric phenotypes of age-related hearing loss from animal models. J Assoc Res Otolaryngol.

[40] Felix H, Pollak A, Gleeson M, Johnsson LG (2002). Degeneration pattern of human first-order cochlear neurons. Adv Otorhinolaryngol.

[41] Wan G, Gómez-Casati ME, Gigliello AR, Liberman MC, Corfas G (2014). Neurotrophin-3 regulates ribbon synapse density in the cochlea and induces synapse regeneration after acoustic trauma. Elife..

[42] Suzuki J, Corfas G, Liberman MC (2016). Round-window delivery of neurotrophin 3 regenerates cochlear synapses after acoustic overexposure. Sci Rep.

[43] Velázquez-Villaseñor L (2000). Temporal bone studies of the human peripheral vestibular system: normative Scarpa’s ganglion cell data. Ann Otol Rhinol Laryngol Suppl.

[44] Robert ME, Linthicum FH (2016). Empirical derivation of correction factors for human spiral ganglion cell nucleus and nucleolus count units. Otolaryngol Head Neck Surg.

[45] R Development Core Team. R: A language and environment for statistical computing. *R Foundation for Statistical Computing* http://www.R-project.org (2016).

