# Supplementary material for: Human Cochlear Histopathology Reflects Clinical Signatures of Primary Neural Degeneration
Source: Sci Rep. 2017 Jul 7;7:4884. doi: 10.1038/s41598-017-04899-9 (PMC5501826; doi:10.1038/s41598-017-04899-9)
Supplement: Supplementary file 1 — Supplementary PDF File [file 41598_2017_4899_MOESM1_ESM.pdf]

Supplementary Information

Human Cochlear Histopathology Reflects Clinical Signatures of Primary Neural Degeneration

Jessica E. Sagers, Lukas D. Landegger, Steven Worthington, Joseph B. Nadol, Jr., Konstantina M. Stankovic

Supplementary Figure S1. Schematic demonstrating the configuration of the linear mixed model (**Fig. 4A**). Four to six hearing threshold observations per patient (depending on which frequencies were clinically examined), color-coded by each individual audiometric test frequency, were graphed vertically on the y-axis at the point along the x-axis representing total neuronal loss for that patient (as percent of age-matched control). In this way, relationships among patients at individual audiometric test frequencies can be examined overall as a function of total neuronal loss.

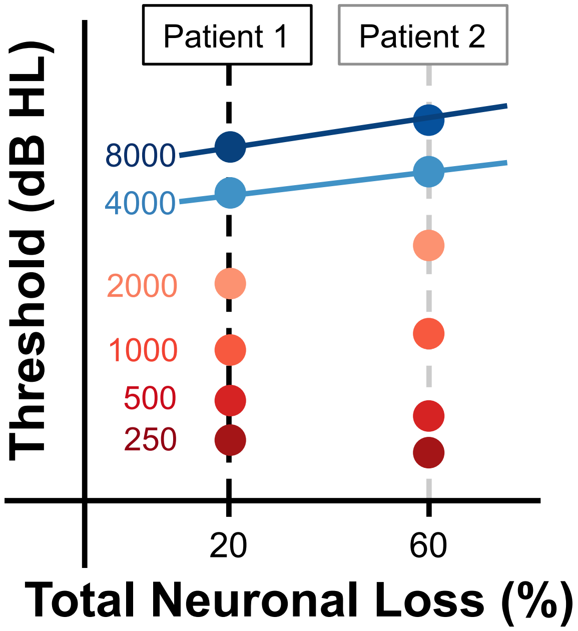

Supplementary Table S1. Summary of predictions for the model depicted in **Fig. 4** by total neuronal loss (Total Loss %) and audiometric test frequency (Test Freq). Predicted means (Fit) are provided with lower and upper bounds of the 95% confidence interval (Lower, Upper).

Total Loss (%)	Test Freq (Hz)	Fit	Lower	Upper
10	250	24.09	8.99	39.20
20	250	30.24	17.71	42.78
30	250	36.39	25.80	46.98
40	250	42.54	32.89	52.20
50	250	48.69	38.68	58.70
60	250	54.84	43.29	66.39
70	250	60.99	47.11	74.86
80	250	67.14	50.48	83.80
90	250	73.29	53.57	93.00
100	250	79.43	56.50	102.37
10	500	25.61	10.53	40.70
20	500	32.15	19.65	44.66
30	500	38.69	28.14	49.25
40	500	45.24	35.63	54.84
50	500	51.78	41.81	61.74
60	500	58.32	46.82	69.82
70	500	64.86	51.03	78.69
80	500	71.40	54.78	88.02
90	500	77.94	58.26	97.62
100	500	84.48	61.58	107.38
10	1000	28.33	13.25	43.41
20	1000	35.22	22.71	47.72
30	1000	42.10	31.55	52.65
40	1000	48.99	39.38	58.59

50	1000	55.87	45.91	65.83
60	1000	62.76	51.26	74.26
70	1000	69.64	55.82	83.47
80	1000	76.53	59.91	93.15
90	1000	83.42	63.74	103.10
100	1000	90.30	67.40	113.20
10	2000	27.04	11.95	42.12
20	2000	34.54	22.03	47.04
30	2000	42.04	31.49	52.59
40	2000	49.54	39.93	59.15
50	2000	57.04	47.08	67.01
60	2000	64.54	53.05	76.04
70	2000	72.05	58.22	85.87
80	2000	79.55	62.93	96.17
90	2000	87.05	67.37	106.73
100	2000	94.55	71.65	117.45
10	4000	45.02	29.94	60.10
20	4000	49.83	37.32	62.34
30	4000	54.64	44.08	65.19
40	4000	59.45	49.84	69.05
50	4000	64.25	54.29	74.22
60	4000	69.06	57.56	80.56
70	4000	73.87	60.04	87.70
80	4000	78.68	62.06	95.30
90	4000	83.49	63.81	103.17
100	4000	88.30	65.40	111.20
10	8000	54.73	39.33	70.14
20	8000	58.96	46.16	71.75

30	8000	63.18	52.38	73.98
40	8000	67.40	57.60	77.19
50	8000	71.62	61.54	81.69
60	8000	75.84	64.28	87.39
70	8000	80.06	66.21	93.91
80	8000	84.28	67.65	100.91
90	8000	88.50	68.81	108.19
100	8000	92.72	69.80	115.63

Supplementary Table S2. Summary of contrasts between audiometric test frequency-specific predictions for the model depicted in **Fig. 4**. SE, standard error; df, degrees of freedom. P-value adjusted by sequential Bonferroni method for 15 tests.

Contrast	Estimate	SE	df	t-ratio	p-value
250-500	-2.79	3.61	141.05	-0.774	0.8810
250-1000	-6.62	3.61	141.05	-1.836	0.3421
250-2000	-7.32	3.61	141.05	-2.030	0.3094
250-4000	-16.59	3.61	141.05	-4.599	0.0001
250-8000	-24.02	3.72	141.05	-6.449	<.0001
500-1000	-3.83	3.57	140.99	-1.074	0.8546
500-2000	-4.53	3.57	140.99	-1.270	0.8253
500-4000	-13.80	3.57	140.99	-3.865	0.0017
500-8000	-21.23	3.69	141.08	-5.756	<.0001
1000-2000	-0.70	3.57	140.99	-0.196	0.8810
1000-4000	-9.97	3.57	140.99	-2.791	0.0538
1000-8000	-17.39	3.69	141.08	-4.717	0.0001
2000-4000	-9.27	3.57	140.99	-2.595	0.0836
2000-8000	-16.69	3.69	141.08	-4.527	0.0001
4000-8000	-7.43	3.69	141.08	-2.014	0.3094

Supplementary Table S3. Summary of predictions for model depicted in **Fig. 5** by total neuronal loss (Total Loss %) and audiometric test frequency, grouped by category (Low/High). Predicted means (Fit) are provided with lower and upper bounds of the 95% confidence interval for slope (Lower, Upper).

Total Loss (%)	Low/High	Fit	Lower	Upper
10	Low	26.31	12.72	39.90
20	Low	33.08	21.83	44.33
30	Low	39.85	30.38	49.33
40	Low	46.63	38.01	55.24
50	Low	53.40	44.46	62.34
60	Low	60.17	49.83	70.51
70	Low	66.95	54.49	79.40
80	Low	73.72	58.73	88.70
90	Low	80.49	62.74	98.25
100	Low	87.27	66.60	107.93
10	High	49.41	35.21	63.61
20	High	53.98	42.21	65.75
30	High	58.56	48.64	68.48
40	High	63.13	54.12	72.15
50	High	67.71	58.38	77.04
60	High	72.29	61.52	83.05
70	High	76.86	63.92	89.81
80	High	81.44	65.87	97.01
90	High	86.01	67.57	104.45
100	High	90.59	69.13	112.06
